# Transitioning Adolescents and Young Adults with Lipid Disorders to Adult Health Care

**DOI:** 10.1007/s11883-024-01244-0

**Published:** 2024-10-02

**Authors:** Christopher Schmitt, Thomas M. Yohannan

**Affiliations:** 1 Department of Pediatrics, Division of Cardiology, Nationwide Children’s Hospital, Columbus, OH USA; 2 Department of Pediatrics, Division of Cardiology, University of Tennessee Health Science Center, Le Bonheur Children’s Hospital, 49 N. Dunlap St, Memphis, TN 38103 USA

**Keywords:** Dyslipidemia, Hypercholesterolemia, Transition, Adolescent, Familial Hypercholesterolemia, FH

## Abstract

**Purpose of Review:**

Pediatric healthcare providers have increasingly become aware of the need for timely and informative transition of adolescents and young adults with chronic medical conditions such as diabetes and cystic fibrosis. However, there is paucity of published data on the importance of and most effective way to transition youth with lipid disorders who are at increased risk of premature cardiovascular disease.

**Recent Findings:**

Evidence shows that atherosclerosis begins at a young age. However, there are no guidelines on the transition of adolescents and young adults with dyslipidemia. In addition, there are conflicting guidelines for lipid management in children versus adults, despite advances in medical pharmacotherapies for dyslipidemia.

**Summary:**

The lack of guidelines for transition and discordant recommendations for management of this vulnerable population places young adults at-risk for worsening of their underlying disease, and premature cardiovascular events.

## Introduction

Atherosclerosis is a chronic condition which, because of lifelong exposure to atherogenic lipids and lipoproteins, begins as fatty streaks which progressed to arterial plaque and thickening of the arterial walls [1, 2]. This process creates endothelial dysfunction and inflammation, which ultimately leads to cardiovascular diseases, such as stroke and myocardial infarction (MI). Globally, atherosclerotic cardiovascular disease (ASCVD) is associated with significant premature morbidity and is the largest cause of mortality in the U.S. (at least 30% of adult mortality attributed to CVD) [3, 4]. Despite being present in 48% of the US adults, atherosclerosis is initially asymptomatic, with clinical signs and symptoms appearing in middle and late adulthood [1, 5].

Atherosclerosis begins in childhood, with evidence of coronary artery thickening and presence of fatty streaks in children as young as 2 years-of-age. In addition to a genetic predisposition, risk factors that adversely influence cardiovascular health, such as a high fat, high carbohydrate diet, lack of exercise, smoking, and alcohol use, first emerge during childhood and adolescence [1, 6–10]. These early changes are underappreciated because of the asymptomatic nature of fatty streaks and atherosclerotic plaques, but become more prominent with age, with incidents of fatal and non-fatal cardiovascular events starting in midlife [11]. In addition to genetic forms of dyslipidemia, studies have shown that children and adolescents with components of metabolic syndrome, have subclinical atherosclerosis. Hence, the American Heart Association (AHA) has recommended frequent screening and when present, tracking of CVD risk factors from childhood to adulthood [12]. Research has shown that identification at a young age and of early interventions, such as lifestyle changes and statins, can slow and often reverse atherosclerotic progression [8, 13]. Although remarkable changes have been accomplished, behavioral and prevention strategies are beginning to plateau in adult populations, renewing interest in primordial prevention with an emphasis on lifestyle changes in youth [3].

## Dyslipidemias

Lipids and lipoproteins are essential for the organization, stability, and structure of cell membranes, as well as being necessary for the synthesis, utilization, and use of certain hormones, vitamins, and bile acids [1].

Disorders affecting lipid and lipoprotein metabolism can be classified according to the most likely underlying etiology [3, 5]:

1. Familial, or genetic
2. Lifestyle
3. Medication
4. Chronic disease

### Genetic Dyslipidemia

These include familial hypercholesterolemia (FH), both homozygous and heterozygous forms, severe hypertriglyceridemia, familial combined hyperlipidemia (FCHL), and elevated lipoprotein(a) [5]. This class of genetic disorder are typically narrow in effect, and often involve only one type of lipid component, for example alterations in low-density lipoprotein cholesterol (LDL-C) or triglycerides (TG) [3, 5].

FH is an autosomal co-dominant disorder that causes dysregulation in LDL metabolism [14, 15]. About 85%-90% of those with FH have variants in the LDL receptor (*LDLR*) gene, however other genes can cause a similar phenotype, including those that encode apolipoprotein B (*APOB*), proprotein convertase subtilisin–kexin type 9 (PCSK9), and LDL receptor adaptor protein 1 (*LDLRAP1*) [16–18]. There are also rare autosomal recessive variants (autosomal recessive hypercholesterolemia), which cause bi-allelic loss-of-function in LDLRAP-1 and a similar phenotype to that of FH, prompting some to refer to this group of disorders as “phenotypic homozygous familial hypercholesterolemia” [14].

Individuals with FH have isolated elevations in LDL-C, and hence an increased lifetime risk of CVD-related events [6, 15]. Those with homozygous FH (HoFH) have two variants and are distinguished by severely elevated LDL-C levels (diagnostic if LDL-C ≥ 500 mg/dL; suspected ≥ 400 mg/dL) and can present with cutaneous and tendon xanthomas during childhood [6, 16]. The most common genetic finding is the presence of a single pathogenic variant ( i.e. heterozygous FH or HeFH), with mild-to-moderate elevation of LDL-C levels (≥ 160 mg/dL). Untreated, men have a 50% risk of a fatal or non-fatal coronary event by age 50 years, and women a 30% risk by age 60 years [19]. Genetic testing is increasingly playing a role in diagnosis and management of these disorders [16].

FCHL is associated with increases in LDL-C and very low-density lipoprotein cholesterol (VLDL-C) levels [3]. The clinical phenotype, often triggered by obesity and insulin resistance, is becoming more prevalent in large part due to the increased prevalence of obesity in youth and adults.

### Lifestyle

A sedentary lifestyle, poor dietary habits, and obesity affect LDL-C, high density lipoprotein cholesterol (HDL-C), and TG levels. Adverse changes in lipids secondary to visceral adiposity is now more prevalent than genetic forms of dyslipidemia [1, 3, 5]. Obesity has become a global epidemic, with subclinical atherosclerosis prevalent in children and adolescents with unhealthy lifestyle-related behaviors [12, 20]. About one third of adults in Western countries suffer from obesity [21]. In addition to its deleterious effects on lipid levels, obesity also increases risk of disease progression, like type 2 diabetes mellitus (T2D), hypertension (HBP), polycystic ovarian syndrome (PCOS), metabolic syndrome (MetS), and fatty liver disease—all of which impede lipid metabolism and promote atherosclerosis, leading to development of CVD and events [5, 20].

### Medications and Chronic Systemic Disease

Derangements in lipid levels are frequently seen in individuals with chronic systemic diseases, and with the use of some medications. Hypothyroidism, nephrotic syndrome, systemic lupus erythematosus, organ transplantation, congenital analbuminemia, anorexia, obstructive liver disease, and lipodystrophies are common systemic diseases that alter lipid levels [5, 16]. Examples of medication known to dysregulate lipid metabolism include protease inhibitors, oral contraceptive pills, cyclosporine and steroids [3, 6, 14].

## Incidence and Global Burden

Estimates of children affected by hyperlipidemia vary by age, being 13–20% in age < 18 years vs age 6–19, the prevalence increasing from 10% three decades ago [4, 6, 22, 23]. Previously, it was estimated that 1:1 million children had HoFH and 1:500 children HeFH. However, recent data shows this to be an underestimate [18]. Current prevalence of HeFH is estimated to be 0.2–0.4%, or 1:200–300 individuals (6.8–8.5 million children worldwide), HoFH 1:200,000–300,000 [13, 15, 16, 18]. FCHL remains the most common genetic dyslipidemia, affecting about 1% of children [24].

Additionally, there is an increased burden of dyslipidemia in children in the absence of known genetic variants. Approximate 7.1%-9.4% of children have increased total cholesterol (> 200 mg/dL), and 10.7% of adolescents (age 12–19) have hypertriglyceridemia (> 150 mg/dL; > 1.7 mmol/L) [6, 23]. This increase is related to the rise of secondary risk factors, like diabetes mellitus (types 1 and 2), obesity, renal disease, and liver disease, with adiposity associated dyslipidemia now the most frequent condition resulting in lipid abnormalities in the pediatric population [1, 6, 24].

## Screening (Table 1)

**Table 1:** Guidelines for Cholesterol Screening in Children

Medical Society	Age (yrs)	Criteria
American Academy of Pediatrics [6, 8]	9–11	Universal screening—Regardless of state of health or family history
	17–2	Universal screening—Regardless of state of health or family history
	≥ 2	Targeted screening—Positive family history for dyslipidemia and/or premature CVD, unknown family history, the presence of risk factors like BMI > 85^th^ percentile, hypertension, cigarette smoking, diabetes mellitus or a high risk medical condition predisposing to hyperlipidemia
European Atherosclerosis Society / European Society of Cardiology [10, 14, 25]	5–11	Universal screening—Regardless of date of health or family history
	< 5	Targeted screening if parents have HeFH or HoFH

There is evidence that early identification and treatment of dyslipidemia reduces the burden of subclinical atherosclerosis and adverse cardiovascular events during adulthood [13]. A major impediment to tackling the global ASCVD burden is the lack of optimal screening of children. For example, out of an estimated 30,000 people worldwide with HoFH, < 5% have been identified, and many of those who have been identified (median age 12 years) have evidence of coronary atherosclerosis or aortic valve disease [14, 15]. Only about 30% of US pediatricians reported adherence to guidelines for universal screening, often due to lack of comfort prescribing statins and/or a lack of access to lipid specialists [4, 11].

## Treatment and Management (Table 2)

**Table 2:** List of Lipid Lowering Medications

Lipid lowering agent	Name, Indication and Age (Yr)	FDA approved for use < 18 years-of-age	Mechanism of action
Statins [1, 6, 13]	Atorvastatin- HeFH (10–17) Fluvastatin- HeFH (10–16) Lovastatin- HeFH (10–17) Pravastatin—HeFH(8–17) Rosuvastatin- HeFH (6–17) Simvastatin- HeFH (10–17) Pitavastatin-HeFH (8–17)	Yes	3-hydroxy-3 methylglutaryl coenzyme A reductase inhibitors
Bile Acid sequestrants [6, 13]	Colesevelam- HeFH (10–17) Colestipol- Primary hypercholesterolemia (7–12) Cholestyramine- Adjunct therapy hypercholesterolemia (6–12)	Colesevelam – Yes Colestipol and Cholestyramine -No	Binds to bile acids and prevents its reabsorption through the intestinal wall
Niacin [13]	Niacin Extended release ≥ 16 Immediate release ≥ 10 Adjunct therapy in hypercholesterolemia	No	
Ezetimibe [6, 13, 26]	Ezetimibe—HoFH (> 10)	Yes	Cholesterol Absorption Inhibitors
Fibric acid derivatives [6, 13]	Fenofibrate Gemfibrozil	No	Peroxisome proliferator-activated receptors (PPARs)activators
PCSK9 inhibitors [13, 17, 27–30]	Alirocumab—HeFH (8–17) Evolocumab—HoFH(10–17)	Evolocumab -Yes Alirocumab—No	Monoclonal antibodies against human proprotein convertase subtilisin/kexin type 9 (PCSK9)
ANGPTL3 inhibitor [13, 26]	Evinacumab—HoFH (5–17)	Yes	Monoclonal antibody against ANGPTL3 (angiopoietin like protein 3)
Inclisiran (siRNA) [13, 31]	Inclisiran- HeFH and HoFH ( trials ongoing in 12–18)	No	Breaks down the messenger (m)RNA that codes for the PCSK9 protein
Microsomal triglyceride transfer protein inhibitor [13, 26]	Lomitapide- HoFH (5–17)	No	Inhibitor of microsomal triglyceride transfer protein (MTP)
Adenosine triphosphate citrate lyase inhibitor [13]	Bempedoic acid (ongoing trials)	No	Inhibitor of ATP citrate lyase enzyme prior to action of HMG Co-A reductase
2’-O-methoxyethyl (2’-MOE) antisense therapeutic oligonucleotide	Volanesorsen	No	Inhibitor of apoC3 by interrupting mRNA translation reducing triglyceride plasma levels

Management of pediatric dyslipidemia is based on the most likely etiology, comorbidities, and the levels of cholesterol and TGs. The cornerstone of management is lifestyle modifications (including diet and exercise) with or without pharmacotherapy. Despite guidelines, dyslipidemia is undertreated in children and adolescents [11].

Because children with HoFH may experience symptomatic CVD and death prior to age 20, aggressive treatment is needed. In addition to lifestyle modifications this includes use of statins, and additional medications like ezetimibe, PCSK9 inhibitors, and lomitapide initiated at the time of diagnosis [15, 16, 27]. Lipoprotein apheresis and consideration of liver transplant are adjunct therapies [14, 15]. Surveillance for ASCVD includes echocardiography, computed tomographic angiograms, and coronary angiography [16, 28].

In those without a genetic variant for APOB, LDLR, LDLRAP1 and PCSK9, testing for other variants that mimic FH, such as sitosterolemia and lysosomal acid lipase deficiency, should be considered. This is important since, if present, these conditions require a different therapeutic approach, such as unique dietary modifications, ezetimibe and enzyme replacement therapies [14].

Youth with HeFH benefit from a statin, with or without ezetimibe, beginning at 8–10 years of age [29]. For secondary causes of hypercholesterolemia, like those associated with obesity and insulin resistance, the decision to initiate pharmacotherapy depends on the persistence of risk factors after a 6 – 12 month trial of lifestyle modifications [3, 5, 11].

### Lifestyle Modifications

The backbone of management is lifestyle modifications—including a heart healthy diet, daily physical activity, and avoidance or cessation of high-risk behaviors like tobacco smoking, vaping and excess alcohol intake [5, 6].

## Transition from Pediatric to Adult Care

The transition from adolescence to adulthood is a crucial stage of development, marked by numerous life changes, increasing autonomy, independence and shifting social structures, which is challenging for those with chronic medical conditions [9]. Transition of care is a planned and purposeful movement of an adolescent, often with chronic medical conditions, from a pediatric-oriented healthcare system to an adult environment. This process can take months to years, depending on the individual, and includes education about their health conditions, knowledge of self-management and a discussion of the skills needed for self-advocacy. This process is enhanced by open communication between the adolescent/family and the health care community, both pediatric and adult. Transition of adolescents with developmental or behavioral issues may require extra time, and the assistance of other healthcare professionals, such as mental health specialists and social workers.

Because a lack of effective and timely transition may result in adverse health outcomes and increased health care costs, there has been an increased focus on improving the transition of care for adolescence and young adults with chronic diseases [32]. This process may involve care coordinators, access to electronic and portable health records, dedicated transition clinics, and use of technology (including mobile and web-based tools, and virtual reality) [9, 33]. Typically, the physical transfer to adult practices occurs in most pediatric practices around 18 years-of-age. Ideally, however, the process begins much earlier, including having the adolescent actively participate in shared decision making, setting goals and objectives for patient education (knowledge about disease, medications, etc.), and empowering the adolescent to make decisions for themselves, arranging future appointments and requesting medication refills [9, 19].

Some pediatric specialties have created disease-specific transition clinics, one of the first being for children with spina bifida 1993 [32]. Other example include pediatric oncology centers which utilized survivorship and/or transitional care plans as part of their routine care, as well as other diseases such as hemophilia, diabetes, juvenile rheumatoid arthritis, sickle cell disease, and cystic fibrosis [34, 35]. In 2008, the American College of Cardiology (ACC) and AHA recommended improvement in organization and access to regional adult congenital heart disease (ACHD) centers, as well as increasing awareness amongst specialists who care for these children. In 2011 the AHA challenged existing programs to include educational initiatives to increase the quality of life, life expectancy, and health literacy of patients [33, 35]. As a result, there have been many ACHD programs and centers created in the U.S. to aid in the care of this transitional population.

However, such clinics and programs are not specifically tailored to address the unique needs of adolescence and young adults at risk of premature CVD, including those with genetic disorders and acquired conditions such as diabetes mellitus, renal disease and autoimmune disease [9]. This, in part, is due to CVD risk factors causing few if any symptoms during child, and a lack of awareness by both the lay and professional community. Furthermore, changing from a pediatric to adult health care environment occurs at a period in life when adolescents and young adults are developing habits and behaviors, both healthy and unhealthy, that impact cardiovascular health, many of which remain throughout their life span [9, 10, 19].

As a growing number of individuals at-risk of premature CVD are identified at a young age, there is a growing need to establish successfully models of transitional care. The growing complexity of medical care, coupled with the increase demand for services and need for personalized care, create challenges. Nonetheless, ensuring the best possible outcomes in this vulnerable population requires careful planning and timely execution. The most successful models for transitional care involves a stepwise approach to educating the adolescent about their disease, goals of therapy and medications, as well as encouraging independent, empowerment and self-efficacy [9].

## Barriers

Commonly encountered barriers to successful transition include the following [19].

1. The adolescent’s…
  - lack of knowledge about their disease(s) and care
  - reluctance to embrace their autonomy and desire to prolong parental involvement in their care
  - competition between life, work, family, social and health care priorities
2. A lack of knowledgeable and experienced adult lipid specialists.
3. Ineffective/incomplete communication between pediatric and adult health care providers
4. Medical insurance

Interviews of individuals with FH and their parents revealed the following challenges [19]:

- The adolescence’s difficulty making independent decisions about a disease with limited signs and symptoms
- The adolescents ability to manage the social stigmata of their disease
- Lack of adequate patient resources

Additionally, disorders of lipid and lipoprotein metabolism in the pediatric population is largely underdiagnosed and undertreated. This, in part, is due to a lack of understanding of guidelines by healthcare professionals. There is also, however, incongruence of the diagnostic and therapeutic recommendations by pediatric versus adult-oriented scientific organizations [10, 11]. Adult guidelines only recommend initiating statin therapy in individuals aged 20–39 who have LDL-C > 190 mg/dL and suggest consideration of statin therapy > 160 mg/dL if there are other risk factors. Pediatric guidelines stratify patients based on risk factors and recommend statin therapy using cut-off values of 130 mg/dL, 160 mg/dL, or 190 mg/dL based on the number of risk factors and comorbidities [6, 9, 12, 19]. National Health and Nutrition Examination Survey (NHANES) estimated 400,000–500,000 adolescents (17–21 years-of-age) would qualify for pharmacotherapy using pediatric guidelines, in contrast to those who would qualify for similar care using adult guidelines [10, 19]. These differences can be confusing to adolescents and healthcare providers alike, and impede effective transition of care [10].

Another important barrier to effective transition is the complicated referral and medication authorization process that varies by state and type of insurance coverage. For example, PCSK9 inhibitors often requires step therapy for insurance approval, necessitating detailed documentation and transfer of documentation to ensure no gaps in coverage [26].

In order to create a successful transition model, pediatric clinics and healthcare providers should strive to increase disease awareness and its potential consequences amongst their adolescents and young adult, encourage autonomy, and how best to work within the healthcare system. Adult clinics, likewise, should assure open communication with pediatric healthcare providers, recognize the gaps and discrepancies in current guidelines, and help support young adults adapt to and successfully navigate the complex healthcare landscape.

Although still limited, there appears to be an increasing number of qualified and experienced adult lipid clinics to ensure that appropriate care and follow-up of pediatric patients. Some practical tips to aid health care providers achieve successful transition of care are listed below [9, 36, 37].

1. Develop a formalized plan for the transition process and discuss it with the adolescent/young adult and their families often.
2. Educate adolescents gradually, increasingly the complexity (appropriate for developmental age) over several visits.
3. Organize and track the patient’s progress.
4. Providing age and diagnosis specific resources (both written and web-based).
5. Leverage technology (electronic medical records and portable health summaries).
6. Address other relevant adolescent issues which may impact compliance.
7. Utilize clinical resources such as coordinators, social workers, and existing transition clinics, when available.
8. Provide appropriate and timely referrals (to both adult general and specialty providers).

Based on interviews of individuals with FH, several suggested interventions were identified [16, 19].

1. Leveraging parents and peers to promote and maintain healthy lifestyles.
2. Setting daily routines.
3. Providing credible external resources.
4. Early engagement of the young adult in medical decision-making.
5. Optimizing the use of genetic testing, especially as the cost burden of these tests decrease. Such knowledge may encourage long-term compliance and willingness to pursue follow-up health care.

## The Transition Process

The transition process is unique to each individual and should be individualized based on the adolescent’s diagnosis, developmental age, comorbidities, and the clinical environment. Ideally, this process should start in early adolescence, at approximately 12 years-of-age, or at time of diagnosis if older. During the course of scheduled visits, adolescents should be taught about their disease and diagnosis, these short- and long-term consequences, specifics of their treatment regimens and educational resources. It is important to receive continuous feedback from the adolescent and family, modifying and tailoring the transition process depending on the feedback. It's also important to measure and record the information the adolescent has learned, to identify gaps in knowledge and skills, and eventually empower the adolescent to transition to a self-directed learner. The adolescent and family should be cognizant of the need for lifelong care, beginning shortly after transition. Barrier should be identified early and potential solutions discussed to assure success.

Approximately 1–2 years prior to transfer, the rationale and process of transition should be discussed. The differences between pediatric and adult oriented health-care systems should be reviewed, and a plan for transition agreed upon, including logistics and timing (the exact age of transfer depends on the adolescent, family, healthcare provider preference, and other factors). A portable healthcare summary should be made available to the adolescence/young adult if not already provided. Adolescents should also learn to take more responsibility for their health and have an introduction to the healthcare landscape. They should be encouraged to make their own appointments, refill medications (with knowledge of how the process works and who to contact), how to contact the clinic/provider with questions or concerns, need to carry a copy of their insurance card, and how to understand their laboratory results.

A few months prior to transfer, confirm the adolescence/young adult’s understanding of the need to establish a follow-up appointment in an adult facility, has a reasonable understanding of current and future insurance options, arrange for coordinators or assistance, if needed (social workers, clinic coordinators, financial assistance), assure an appropriate referral has been made, assure the patient knows how to access their healthcare records, and be sure a portable summary has been created and given to the patient and the referred physician. Additionally, the adolescence/young adult should be made aware of the need for a properly execute HIPAA waiver if they wish the parents or others to have access to medical information after age 18, and a medical power of attorney should be considered. If desirable, a follow-up visit can be made after the transfer to ensure that the adolescent has no further questions, was able to make a follow-up appointment, and for the health care provider to adjust the success of the transition process based upon the lessons learned.

## Conclusions

Thanks to improved awareness and access to healthcare, a growing number of children with chronic medical conditions survive into adulthood. The benefits of effective and timely transitional from the pediatric to the adult health care environment has been demonstrated in many conditions, such as diabetes and cystic fibrosis. While equally beneficial, this need is less apparent in adolescents and young adults with lipids disorders who are at-risk of premature CVD. More research is needed to identify the best means of transitional care. To ensure the best outcomes, health care professionals are encouraged to develop individualized transition plans and identify qualified colleagues within their community to provide ongoing care for this vulnerable population.

## Data Availability

No datasets were generated or analysed during the current study.

## References

[1] Fiorentino R, Chiarelli F. Statins in children, an update. Int J Mol Sci. 2023;24(2):1366. 10.3390/ijms24021366. Published 2023 Jan 10. 36674877 10.3390/ijms24021366 PMC9862804

[2] Ratajczak M, Skrypnik D, Bogdański P, et al. Effects of endurance and endurance-strength training on endothelial function in women with obesity: a randomized trial. Int J Environ Res Public Health. 2019;16(21):4291. 10.3390/ijerph16214291. Published 2019 Nov. 31694237 10.3390/ijerph16214291 PMC6862069

[3] Leopold S, Zachariah JP. Pediatric lipid disorders. Pediatr Ann. 2021;50(3):e105–12. 10.3928/19382359-20210218-01. 34038650 10.3928/19382359-20210218-01 PMC8544611

[4] Ashraf AP, Kohn B, Wilson DP. Improving long-term outcomes of youth with lipid abnormalities-expanding the role of pediatric endocrinologists. J Clin Endocrinol Metab. 2019;104(10):4421–6. 10.1210/jc.2019-00150. 31120509 10.1210/jc.2019-00150

[5] Lazarte J, Hegele RA. Pediatric dyslipidemia-beyond familial hypercholesterolemia. Can J Cardiol. 2020;36(9):1362–71. 10.1016/j.cjca.2020.03.020. 32640212 10.1016/j.cjca.2020.03.020

[6] Stewart J, McCallin T, Martinez J, Chacko S, Yusuf S. Hyperlipidemia. Pediatr Rev. 2020;41(8):393–402. 10.1542/pir.2019-0053. 32737252 10.1542/pir.2019-0053

[7] Burlutskaya AV, Tril VE, Polischuk LV, Pokrovskii VM. Dyslipidemia in pediatrician’s practice. Rev Cardiovasc Med. 2021;22(3):817–34. 10.31083/j.rcm2203088. 34565080 10.31083/j.rcm2203088

[8] Khoury M, Rodday AM, Mackie AS, et al. Pediatric lipid screening and treatment in Canada: practices, attitudes, and barriers. Can J Cardiol. 2020;36(9):1545–9. 10.1016/j.cjca.2020.05.035. 32502521 10.1016/j.cjca.2020.05.035

[9] Chung RJ, Mackie AS, Baker A, de Ferranti SD. Cardiovascular risk and cardiovascular health behaviours in the transition from childhood to adulthood. Can J Cardiol. 2020;36(9):1448–57. 10.1016/j.cjca.2020.05.041. 32585325 10.1016/j.cjca.2020.05.041

[10] Gooding HC, Rodday AM, Wong JB, et al. Application of pediatric and adult guidelines for treatment of lipid levels among us adolescents transitioning to young adulthood. JAMA Pediatr. 2015;169(6):569–74. 10.1001/jamapediatrics.2015.0168. 25845026 10.1001/jamapediatrics.2015.0168

[11] Kim GK, Yee JK, Bansal N. Algorithms for treating dyslipidemia in youth. Curr Atheroscler Rep. 2023;25(8):495–507. 10.1007/s11883-023-01122-1. 37523052 10.1007/s11883-023-01122-1

[12] Grundy SM, Stone NJ, Bailey AL, et al. AHA/ACC/AACVPR/AAPA/ABC/ACPM/ADA/AGS/APhA/ASPC/NLA/PCNA Guideline on the management of blood cholesterol: a report of the American College of Cardiology/American Heart Association Task Force on Clinical Practice Guidelines [published correction appears in Circulation. 2019 Jun 18;139(25):e1182-e1186] [published correction appears in Circulation. 2023 Aug 15;148(7):e5]. Circulation. 2019;139(25):e1082-e1143. 10.1161/CIR.0000000000000625. 10.1161/CIR.0000000000000625 PMC7403606 30586774

[13] Choudhari P, Patni N. Updates in the management of pediatric dyslipidemia. Curr Opin Lipidol. 2023;34(4):156–61. 10.1097/MOL.0000000000000879. 36942877 10.1097/MOL.0000000000000879

[14] Cuchel M, Raal FJ, Hegele RA, et al. 2023 update on European atherosclerosis society consensus statement on homozygous familial hypercholesterolaemia: new treatments and clinical guidance. Eur Heart J. 2023;44(25):2277–91. 10.1093/eurheartj/ehad197. 37130090 10.1093/eurheartj/ehad197 PMC10314327

[15] Tromp TR, Hartgers ML, Hovingh GK, et al. Worldwide experience of homozygous familial hypercholesterolaemia: retrospective cohort study. Lancet. 2022;399(10326):719–28. 10.1016/S0140-6736(21)02001-8. 35101175 10.1016/S0140-6736(21)02001-8 PMC10544712

[16] Mainieri F, Tagi VM, Chiarelli F. Recent advances on familial hypercholesterolemia in children and adolescents. Biomedicines. 2022;10(5):1043. 10.3390/biomedicines10051043. Published 2022 Apr. 35625781 10.3390/biomedicines10051043 PMC9139047

[17] Santos RD, Ruzza A, Hovingh GK, et al. Evolocumab in pediatric heterozygous familial hypercholesterolemia. N Engl J Med. 2020;383(14):1317–27. 10.1056/NEJMoa2019910. 32865373 10.1056/NEJMoa2019910

[18] Harada-Shiba M, Kastelein JJP, Hovingh GK, et al. Efficacy and safety of Pitavastatin in children and adolescents with familial hypercholesterolemia in Japan and Europe. J Atheroscler Thromb. 2018;25(5):422–9. 10.5551/jat.42242. 29187694 10.5551/jat.42242 PMC5945555

[19] Sliwinski SK, Gooding H, de Ferranti S, et al. Transitioning from pediatric to adult health care with familial hypercholesterolemia: listening to young adult and parent voices. J Clin Lipidol. 2017;11(1):147–59. 10.1016/j.jacl.2016.11.001. 28391881 10.1016/j.jacl.2016.11.001 PMC6547361

[20] Brzeziński M, Metelska P, Myśliwiec M, Szlagatys-Sidorkiewicz A. Lipid disorders in children living with overweight and obesity- large cohort study from Poland. Lipids Health Dis. 2020;19(1):47. 10.1186/s12944-020-01218-6. Published 2020 Mar 16. 32178670 10.1186/s12944-020-01218-6 PMC7076982

[21] Valenzuela PL, Santos-Lozano A, Saco-Ledo G, Castillo-García A, Lucia A. Obesity, cardiovascular risk, and lifestyle: cross-sectional and prospective analyses in a nationwide Spanish cohort. Eur J Prev Cardiol. 2023;30(14):1493–501. 10.1093/eurjpc/zwad204. 37317985 10.1093/eurjpc/zwad204

[22] Wójcik M. Lipid disorders in children - an underestimated problem. Pediatr Endocrinol Diabetes Metab. 2022;28(4):241–4. 10.5114/pedm.2022.122050. 36734389 10.5114/pedm.2022.122050 PMC10214930

[23] Guirguis-Blake JM, Evans CV, Coppola EL, Redmond N, Perdue LA. Screening for lipid disorders in children and adolescents: updated evidence report and systematic review for the us preventive services task force. JAMA. 2023;330(3):261–74. 10.1001/jama.2023.8867.37462700 10.1001/jama.2023.8867

[24] Ashraf AP, Sunil B, Bamba V, et al. Case studies in pediatric lipid disorders and their management. J Clin Endocrinol Metab. 2021;106(12):3605–20. 10.1210/clinem/dgab568. 34363474 10.1210/clinem/dgab568 PMC8787854

[25] Expert Panel on Integrated Guidelines for Cardiovascular Health and Risk Reduction in Children and Adolescents, National Heart, Lung, and Blood Institute. Expert panel on integrated guidelines for cardiovascular health and risk reduction in children and adolescents: summary report. Pediatrics. 2011;128 Suppl 5(Suppl 5):S213-S256. 10.1542/peds.2009-2107C. 10.1542/peds.2009-2107C PMC4536582 22084329

[26] Butt WZ, Yee JK. The role of non-statin lipid-lowering medications in youth with hypercholesterolemia. Curr Atheroscler Rep. 2022;24(5):379–89. 10.1007/s11883-022-01013-x. 35344138 10.1007/s11883-022-01013-x

[27] Bruckert E, Caprio S, Wiegman A, et al. Efficacy and safety of alirocumab in children and adolescents with homozygous familial hypercholesterolemia: phase 3, multinational open-label study. Arterioscler Thromb Vasc Biol. 2022;42(12):1447–57. 10.1161/ATVBAHA.122.317793. 36325897 10.1161/ATVBAHA.122.317793 PMC9750107

[28] Gidding SS, Hegele RA. Introducing personalized medicine into pediatric homozygous familial hypercholesterolemia care. Arterioscler Thromb Vasc Biol. 2022;42(12):1458–60. 10.1161/ATVBAHA.122.318598. 36325896 10.1161/ATVBAHA.122.318598

[29] Santos RD, Ruzza A, Hovingh GK, et al. Paediatric patients with heterozygous familial hypercholesterolaemia treated with evolocumab for 80 weeks (HAUSER-OLE): a single-arm, multicentre, open-label extension of HAUSER-RCT. Lancet Diabetes Endocrinol. 2022;10(10):732–40. 10.1016/S2213-8587(22)00221-2. 36075246 10.1016/S2213-8587(22)00221-2

[30] Santos RD, Wiegman A, Caprio S, et al. Alirocumab in pediatric patients with heterozygous familial hypercholesterolemia: a randomized clinical trial. JAMA Pediatr. 2024;178(3):283–93. 10.1001/jamapediatrics.2023.6477. 38315470 10.1001/jamapediatrics.2023.6477 PMC10845038

[31] Reijman MD, Schweizer A, Peterson ALH, et al. Rationale and design of two trials assessing the efficacy, safety, and tolerability of inclisiran in adolescents with homozygous and heterozygous familial hypercholesterolaemia. Eur J Prev Cardiol. 2022;29(9):1361–8. 10.1093/eurjpc/zwac025. 35175352 10.1093/eurjpc/zwac025

[32] Hopson B, Eckenrode M, Rocque BG, et al. The development of a transition medical home utilizing the individualized transition plan (ITP) model for patients with complex diseases of childhood. Disabil Health J. 2023;16(2):101427. 10.1016/j.dhjo.2022.101427. 36621354 10.1016/j.dhjo.2022.101427

[33] Kieu V, Sumski C, Cohen S, Reinhardt E, Axelrod DM, Handler SS. The use of virtual reality learning on transition education in adolescents with congenital heart disease. Pediatr Cardiol. 2023;44(8):1856–60. 10.1007/s00246-023-03292-w. 37676275 10.1007/s00246-023-03292-w

[34] Goldfarb M, Franco AT. Survivorship, quality of life, and transition to adult care for pediatric and adolescent thyroid cancer survivors. Thyroid. 2022;32(12):1471–6. 10.1089/thy.2022.0407. 36193568 10.1089/thy.2022.0407

[35] Hays L. Transition to adult congenital heart disease care: a review. J Pediatr Nurs. 2015;30(5):e63–9. 10.1016/j.pedn.2015.01.025. 25704989 10.1016/j.pedn.2015.01.025

[36] Durrington P. Dyslipidaemia. Lancet. 2003;362(9385):717–31. 10.1016/S0140-6736(03)14234-1. 12957096 10.1016/S0140-6736(03)14234-1

[37] Stone NJ, Robinson JG, Lichtenstein AH, et al. ACC/AHA guideline on the treatment of blood cholesterol to reduce atherosclerotic cardiovascular risk in adults: a report of the American College of Cardiology/American Heart Association Task Force on Practice Guidelines [published correction appears in J Am Coll Cardiol. 2014 Jul 1;63(25 Pt B):3024–3025] [published correction appears in J Am Coll Cardiol. 2015 Dec 22;66(24):2812]. J Am Coll Cardiol. 2014;63(25 Pt B):2889–2934. 10.1016/j.jacc.2013.11.002. 10.1016/j.jacc.2013.11.002 24239923

